# Drug use evaluation in pregnant women attending antenatal care in Shashemene Referral Hospital, Oromia Regional State, Ethiopia

**DOI:** 10.1177/2050312120959178

**Published:** 2020-09-18

**Authors:** Gudeta Duga Geresu, Dirirsa Tashome Sondesa, Tadele Mekuriya Yadesa, Andrew G Mtewa, Bontu Aschale Abebe

**Affiliations:** 1 Department of Pharmacy, Ambo University, Ambo, Ethiopia; 2 Department of Chemistry, Malawi University of Science and Technology, Thyolo, Malawi; 3 Department of Midwifery, Ambo University, Ambo, Ethiopia

**Keywords:** Drug use, pregnant women, Shashemene, Ethiopia

## Abstract

**Objectives:**

The main aim of this study was to estimate relative proportions of medication use according to different pregnancy risk categories (A, B, C, D, X) among pregnant women attending antenatal care (ANC) visits at Shashemene Referral Hospital.

**Methods:**

A hospital-based retrospective cross-sectional study was conducted at Shashemene referral hospital from February 2016 to February 2017. Structured data collection form was used to capture data from patient medication cards. SPSS version 16.0 was used to analyze the results after entering and importing from MS-Excel.

**Results:**

A total of 317 pregnant women cards were collected and assessed during the study period in May, 2017. Most, 208(65.6%), of the pregnant women were in their second trimester of pregnancy followed by third trimester, 78(24.6%). Tetanus prevention in pregnancy, 274(86.4%), was the most common reason for drug use. Number of medications prescribed was highest, 384(68.2%), in second trimester followed by third trimester, 130(23.1%). More than half, 305(54.2%), of the drugs prescribed were under category C, tetanus toxoid (TT) vaccine alone accounting for 274 (89.8%) of them, followed by 36.8% from category A.

**Conclusion:**

Vaccines, vitamins and minerals were the most frequently prescribed medications. The overall drug use condition during pregnancy in this study was inappropriate as more than half of the prescribed medications were from category C. On the other hand, category X medications were not prescribed.

## Background

Physiological changes during pregnancy quite often require the need for drug therapy which is not always safe. Among the most notorious examples is Thalidomide use for anxiety, insomnia and as an anti-emetic, and the use of which resulted in phocomelia and other congenital anomalies in thousands of children exposed in utero.^1^ Among the crucial outcomes of Evaluation of drug use during pregnancy include the following: the potential risk of harmful effects to fetus, physiological changes resulting in pharmacokinetic and pharmacodynamics changes, and the high drug-utilization patterns during pregnancy.^2^

The use of medications among expecting mothers has often been associated with significant problems in ANC due to the potential fetal risk associated with the use. According to drug utilization studies, the prevalence of drug use during pregnancy is high, varying from 44% to 99% in different areas across the world. In the treatment process of common complaints during pregnancy, a huge fraction of unnecessary use of drugs has been extensively recounted in most developing countries.^3^

In line with endeavors to promote safe use of medications during pregnancy, the US Food and Drugs Administration (FDA) categorized drugs into five pregnancy risk categories: A, B, C, D, and X; among these groups, D and X medications are those with available evidence of significant harm in pregnancy.^4^ Category-A drugs are those with no risk to the fetus from well-controlled human studies. For drugs in category B, no evidence of risk from animal studies has been documented, but there are no controlled studies in pregnant women. Category C drugs are those associated with harm to fetus in animal studies but no adequate and well-controlled studies in pregnant women. For category D drugs, there is well-documented human fetal risk, and these should be generally avoided except occasional use when the maternal benefit outweighs the fetal risk. However, drugs with classification X are associated with prominent fetal harm and are contraindicated in pregnancy.^5^

Despite limited information on its safety, drug use in pregnancy is common. Supplementary drug treatment like iron, folic acid, calcium, and vitamins are prescribed commonly to improve overall nutritional status of mother and fetus. In addition, drugs may also be used for acute conditions not related to pregnancy or pre-existing chronic conditions such as diabetes, hypertension or epilepsy or to treat pregnancy-related disorders such as pregnancy induced hypertension and gestational diabetes. Therefore, adequate knowledge and more sensible prescription of drugs are crucial among pregnant women.^6^

Currently, drug use evaluation studies mainly involve assessing disease prevalence, drug consumption, rational use of drugs and adherence to evidence-based guidelines.^7^ The primary objective of a drug use evaluation study is to promote safe and effective use of medicines. Accordingly, such studies can potentially contribute in suggesting the right strategies and ways to improve prescribing practices.^8^ An international investigation sponsored by the World Health Organization (WHO) showed that pregnant women take an average of three prescribed drugs during pregnancy.^9^ Approximately 2%–5% of all live births are associated with congenital birth defects and about 1%–3% of all live births with a congenital defect are attributed to medication use by the mothers during pregnancy.^10^

During pregnancy, unnecessary use of drugs has been commonly identified in many developing countries. Self-medication is widely practiced by pregnant women to manage minor pregnancy-related conditions such as back pain, headache, heartburn, nausea, and vomiting.^11^ In these countries, the prevalence of drug use during pregnancy and their pregnancy risk categorization has been largely overlooked.^12^ However, studies from developed countries have indicated importance of ensuring rational prescribing during pregnancy.^13^ Because of the limited knowledge and inconsistencies about the use of drugs during pregnancy, the extensive drug use by pregnant women is an important public health issue.^14,15^ The main aim of this study was to estimate proportions of medication use from different pregnancy risk categories (A, B, C, D, and X) among pregnant women attending ANC visits at Shashemene Referral Hospital.

## Methods

### Study area

This study was retrospectively conducted from February, 2016 to February, 2017 at Shashemene Referral Hospital (SRH), which is found in Kuyera town. It is one of the oldest hospitals of the region; currently serving an estimated 1.2 million people. Geographically, it is located in Kuyera town about 238 km south of the capital city of Addis Ababa. All of the pregnant women in the catchment of the hospital were encouraged by the local health department to have four regular visits during each ANC cycle and all ANC services are completely free of charge in an attempt to improve access by those with low income or women with little awareness about the services. Additional visits were also advised if any complaint is felt by the clients amid the regular follow-up visits. Data were collected from 15 to 30 May 2017.

### Study design

A retrospective cross-sectional study design was employed for drug use evaluation of pregnant women attending ANC at SRH.

### Study populations

All patient cards of pregnant women who visited SRH, attended ANC and used at least one drug were included as the target population in this study.

### Eligibility criteria

Eligible patient cards included those belonging to women who had been already enrolled in ANC program in the hospital and those who were prescribed with at least one medication during the follow-up visits under the current ANC cycle. *P*atient cards *with incomplete or illegible* medication charts with lacking the required drug information were excluded.

## Sample size determination and sampling technique

### Sample size determination

Sample size was calculated using formula for a single population proportion. Since there were no previous studies on the proportion of medication used from each of the different pregnancy risk categories (A, B, C, D or X), the estimate of maximum sample size was calculated from 50% proportion at 95% confidence level and 5% tolerable margin of error

n=((Za2)2×p×(1−p))D2=((1.96)2×0.50×(1−0.50))((0.05)2)=384

The finite population was 1800 patient cards of pregnant women attending antenatal care (ANCs) in SRH all of which were potential participants.

Since the study population was less than 10,000, the final sample size (*n_f_*) was calculated by correction formula for finite population as follows

$$n_{f}=\frac{n}{1+\frac{n}{N}}$$

where *N* is the original sample size, *n_f_* is the final sample size, *Z* is the standard normal value at CI of 95% which is 1.96, *P* is the estimate of prevalence rate, and *D* is the margin of error.

Therefore, nf = $\frac{\left(384*1800\right)}{\left(384+1800\right)}$ = 317 medication cards of pregnant women following ANC were taken as a final sample size.

### Sampling technique

Systematic random sampling technique was used as follows by first numbering patient cards in ascending order from 1 to 1800 using the patient card numbers assigned by the hospital during first enrolment to the hospital.

Second, sampling interval, *K*, was determined K = $\frac{\left(\mathrm{Total}\;\mathrm{population}\right)}{\left(\mathrm{Determined}\;\mathrm{size}\right)},$ K = $\frac{1800}{317}$ ≅ 5

Then, from 1, 2, 3, 4 and 5, number 5 was chosen using random number method; thus, starting with 5 every fifth patient card was included; 5, 10, 15, 20 . . . until the target sample size of 317 was reached.

## Study variables

### Independent variables

- Age
- Trimester of pregnancy
- Duration on ANC
- The prescriber’s knowledge and experience
- Drug information service

### Dependent variables

- Drug use
- Potential teratogenicity

## Data collection and analysis

### Data collection

Data on age of participant, medications given and gestational age were collected using a structured data collection form which was used to extract pertinent information from patient medication files (Supplemental Material).

### Statistical analysis

The collected data were sorted and classified in accordance to US FDA risk classification for pregnancy and analyzed using SPSS version 16.0.

### Data quality control

The data collectors were trained on the use of the data collection tool and on how to review the patient cards before the data collection started. The collected data were checked for its completeness, accuracy, and clarity daily by principal investigator.

## Results

### Participants’ data

The medication cards of a total of 317 pregnant women were studied during the study period. More than half, 178(56.2%), of the subjects were under 15–24 age group followed by 25–34 years, 126 (39.7%). Most, 208 (65.6%), of the pregnant women were in their second trimester of pregnancy followed by third trimester, 78(24.6%; Table 1).

**Table 1.** Age group and gestational age of pregnant women attending ANC in SRH, February 2016 to February 2017.

Variables	Sub-division of variable	Frequency	Percentage
**Age in year**	15–24	178	56.2
	25–34	126	39.7
	35–44	13	4.1
**Gestational age (weeks)**	Second trimester	208	65.6
	Third trimester	78	24.6
	First trimester	31	9.8

ANC: antenatal care; SRH: Shashemene Referral Hospital.

### Reasons for drug use in pregnant women

Tetanus prevention in pregnancy, 274(86.4%), was the most common reason for drug use followed by prevention of anemia, vitamin deficiency and the treatment of anemia 212(66.9%). Genitourinary infections accounted for 23 (7.3%), whereas pain killer, de-worming and respiratory infection accounted for 19 (5.9%), 15 (4.7%) and 7 (2.2%), respectively (Table 2).

**Table 2.** Reasons for drugs use in pregnant women attending ANC at SRH, February 2016 to February 2017.

S. No.	Reason for drug use	Frequency	Percentage
1	Tetanus prevention	274	86.4
2	Prevention and treatment of anemia, vitamin deficiency	212	66.9
3	Treatment of genitourinary infections	23	7.3
4	As pain killer	19	5.9
5	De-worming	15	4.7
6	Treatment of respiratory infections	7	2.2
7	Treatment of gastrointestinal infections	6	1.9
8	To suppress nausea/vomiting	6	1.9
9	Treatment of tonsillopharyngitis	1	0.3

ANC: antenatal care; SRH: Shashemene Referral Hospital.

### Drugs used and trimester of pregnancy

The number of medications used was highest, 384(68.2%), in second trimester followed by third trimester 130(23.1%). Vaccines were the most commonly used drugs, 274 (48.7%), followed by minerals and vitamins, 212 (37.6%; Table 3).

**Table 3.** Drugs used and gestational age of pregnant women attending ANC at SRH February, 2016 to February, 2017.

Class of drugs	Name of drugs	Frequency of drugs in each trimester			Total 563 (100%)
		First trimester 49 (8.7%)	Second trimester 384 (68.2%)	Third trimester 130 (23.1%)	
Vaccine	TT	17	211	46	274 (48.7%)
Minerals and vitamins	Iron/folic acid	26	131	55	212 (37.6%)
Analgesics	Paracetamol, Diclofenac, Ibuprofen		16	3	19 (3.4%)
Antacid	Cimitidine, Omeprazole		2	2	4 (0.7%)
Antibiotics	Amoxicillin, Cephalexin, Augmentin, Doxycycline, Chloramphenicol, Metronidazole, Ciprofloxacin	2	22	9	33 (5.9%)
Anti-emetics	Chlorpromazine, Metoclopramide	4	2		6 (1.1%)
Antihelmentics	Mebendazole			15	15 (2.6%)
Total		49	384	130	563 (100%)

ANC: antenatal care; SRH: Shashemene Referral Hospital; TT: tetanus toxoid.

### FDA risk category of drugs used in pregnant women

More than half, 305(54.2%), of the drugs used by pregnant women were under category C; tetanus toxoid (TT) vaccine accounting for 274 (89.8%). Among these, nearly three fourth, 215(70.5%), were used during second trimester. Category A drugs, 207(36.8%), were the second most commonly used drugs followed by category B, 50(8.9%). No drug under category X was given to pregnant women in this study (Table 4).

**Table 4.** FDA risk category of drugs used in pregnant women at SRH, February 2016 to February 2017.

FDA category	First trimester	Second trimester	Third trimester	Total	Drugs used
A	26	127	54	207 (36.8%)	Iron/folic acid
B	6	33	11	50 (8.9%)	Amoxicillin Cephalexin Augmentin Cimetidine Metronidazole Paracetamol Ibuprofen Metoclopramide
C	30	215	60	305 (54.2%)	TT Tetracycline Chloramphenicol Ciprofloxacin Chlorpromazine Diclofenac Mebendazole Omeprazole
D	0	1	0	1 (0.2%)	Doxycycline
Total	62	376	125	563 (100%)	As listed above

FDA: US Food and Drugs Administration; SRH: Shashemene Referral Hospital; TT: tetanus toxoid.

## Discussion

According to this study, the majority (56.2%) of pregnant women were in the age group of 15–24 years followed by 25–34 years. In this study, TT vaccination was the most frequently used (48.7%) medication during pregnancy followed by Iron/folic acid (37.6%), Antibiotics (5.9%) and Analgesics (3.4%). The use of antibiotics by only 5.9% women was much lower compared to a study from India^16^ in which metronidazole was used by 26.8% and ceftriaxone by 19.4%; as well as study done in Jazan, Saudi Arabia where the use of antibiotics was 19.9%. This variation is probably because the later was conducted at inpatient setting. However, the high rate of use of Iron/Folic acid (69.9%) in the current study is comparable to that of a study conducted on expecting mothers in Jazan, Saudi Arabia in which Iron/Folic acid was used by 82.9%.^17^ The significant use of antibiotics for treatment of urinary tract infections (7.3%) is comparable to a study conducted in the Brazil where most of the antibiotics used were to treat urinary tract infection (UTI).^18^

In this study, majority of drugs were from category C (54.2%), followed by category A (36.8%), category B (8.9%) and D (0.33%). The proportion of using category A medications in this study (36.8%) was much higher than the other similar studies; 15.4% from Rajahmundry^19^ and 14.0% from Brazil.^18^ This can be attributed to the higher rate of iron/folic acid use during ANC as compared to the other studies. On the contrary, the proportion of using category B medications of 8.9% is comparable to 9.1% from a study Rajahmundry^19^ but much lower than 37.2% from the study in Brazil^18^ as well as 26.3% from a study in Jimma, Ethiopia.^5^ This difference might be due to more stringent adherence to Standard Treatment Guidelines (STG) among prescribers in the latter ones.

The proportion of using category C medications was comparable to the proportion of 53.11% from a study done on pregnant women in GSL Medical College and General Hospital, Rajahmundry.^19^ On the other hand, the proportion of using category C (54.2%) was significantly higher than 39.5% from a study in Brazil^18^ probably because of lower previous TT vaccinations in the current study, leading to the higher proportion of the use of TT during pregnancy, accounting for 274 (89.8%) of all category C medications, and a possibly more sophisticated health care system, including a more coordinated health care teams and availability of safer medications, in Brazil, a middle-income county. The occurrence of contraindicated medicines was desirably low.

Similar to a study from Brazil^18^ and another study from India,^1^ no woman was prescribed Category X drug during the current study. This was also comparable to the findings from other studies including Ethiopia,^20^ Netherland^21^ and Finland.^22^

### Limitations of the study

The important limitations encountered during the current study included unavailability of gestational age for some patients, incomplete recording of medications prescribed from other facilities, and the gap in identifying over-the-counter use of conventional as well as herbal medications used by the patients as patient files were the sole sources of the medications used.

## Conclusion and recommendations

### Conclusion

The overall drug use during pregnancy in this study was significantly inappropriate and irrational showing inconsistencies in adhering to STG. Excluding vaccinations, minerals and vitamins were the highest drugs used followed by antibiotics and analgesic drugs. Majority of the drug used were FDA category C, followed by category A and category B. None of the drugs were used from category X which have potential fatal harm to the fetus.

### Recommendations

We recommended that there should be intensive assessment of pregnant women depending on the FDA risk category, gestational period or trimester and the risk benefit balance of a drug before it is prescribed. Drug information center in SRH should play great role in promoting rational drug use for pregnant women in line with national STG. In addition, the hospital pharmacist should work with other health professionals’ team to develop, implement, and deliver drug information and monitor a therapeutic plan to achieve optimal care for all pregnant women.

## Figures and Tables

**Table:** 

Gestational age (weeks)	Trimester	Indication	Drug name	Dosage form	Route of administration	Drug class	Brand name	US FDA risk category
								
								
								
								
								
								

## Supplementary material

**Data Collection Form**

Age (Years) a. 15-24 b. 25-34 c. 35-44 d. >=45.
